# Color Discrimination Is Affected by Modulation of Luminance Noise in Pseudoisochromatic Stimuli

**DOI:** 10.3389/fpsyg.2016.01006

**Published:** 2016-07-06

**Authors:** Iñaki Cormenzana Méndez, Andrés Martín, Teaire L. Charmichael, Mellina M. Jacob, Eliza M. C. B. Lacerda, Bruno D. Gomes, Malinda E. C. Fitzgerald, Dora F. Ventura, Luiz C. L. Silveira, Beatriz M. O'Donell, Givago S. Souza

**Affiliations:** ^1^Departamento de Luminotecnia, Luz y Visión “Ing. Herberto C. Bühler”, Facultad de Ciencias Exactas y Tecnología, Universidad Nacional de TucumánTucumán, Argentina; ^2^Department of Natural Science, Christian Brothers UniversityMemphis, TN, USA; ^3^Instituto de Ciências Biológicas, Universidade Federal do ParáBelém, Brazil; ^4^Núcleo de Medicina Tropical, Universidade Federal do ParáBelém, Brazil; ^5^Department of Biology, Christian Brothers UniversityMemphis, TN, USA; ^6^Department of Anatomy and Neurobiology, University of Tennessee Health Science CenterMemphis, TN, USA; ^7^Department of Experimental Psychology, Instituto de Psicologia, Universidade de São PauloSão Paulo, Brazil; ^8^Universidade do CeumaSão Luís, Brazil

**Keywords:** color vision, pseudoisochromatic stimulus, color-luminance interaction, Cambridge Color Test, color discrimination thresholds, reaction time

## Abstract

Pseudoisochromatic stimuli have been widely used to evaluate color discrimination and to identify color vision deficits. Luminance noise is one of the stimulus parameters used to ensure that subject's response is due to their ability to discriminate target stimulus from the background based solely on the hue between the colors that compose such stimuli. We studied the influence of contrast modulation of the stimulus luminance noise on threshold and reaction time color discrimination. We evaluated color discrimination thresholds using the Cambridge Color Test (CCT) at six different stimulus mean luminances. Each mean luminance condition was tested using two protocols: constant absolute difference between maximum and minimum luminance of the luminance noise (constant delta protocol, CDP), and constant contrast modulation of the luminance noise (constant contrast protocol, CCP). MacAdam ellipses were fitted to the color discrimination thresholds in the CIE 1976 color space to quantify the color discrimination ellipses at threshold level. The same CDP and CCP protocols were applied in the experiment measuring RTs at three levels of stimulus mean luminance. The color threshold measurements show that for the CDP, ellipse areas decreased as a function of the mean luminance and they were significantly larger at the two lowest mean luminances, 10 cd/m^2^ and 13 cd/m^2^, compared to the highest one, 25 cd/m^2^. For the CCP, the ellipses areas also decreased as a function of the mean luminance, but there was no significant difference between ellipses areas estimated at six stimulus mean luminances. The exponent of the decrease of ellipse areas as a function of stimulus mean luminance was steeper in the CDP than CCP. Further, reaction time increased linearly with the reciprocal of the length of the chromatic vectors varying along the four chromatic half-axes. It decreased as a function of stimulus mean luminance in the CDP but not in the CCP. The findings indicated that visual performance using pseudoisochromatic stimuli was dependent on the Weber's contrast of the luminance noise. Low Weber's contrast in the luminance noise is suggested to have a reduced effect on chromatic information and, hence, facilitate desegregation of the hue-defined target from the background.

## Introduction

Pseudoisochromatic stimuli have been used for color vision evaluation since the nineteenth century (Stilling, 1877). Ishihara plates and Cambridge Color Test (CCT) are two well-known examples of a widely varied of color vision tests that employ pseudoisochromatic patterns. These patterns comprise mosaics with elements of different sizes (size noise) and different luminance (luminance noise). Each pattern is composed by a target and a field that differ from each other by their chromaticities. The chromaticity difference is supposed to be the only cue that subjects use to identify the presence of the target amid the field. Pseudoisochromatic tests are of clinical importance to diagnose color vision deficiencies (Regan et al., 1994), to collect normative data (Ventura et al., 2003; Paramei, 2012; Paramei and Oakley, 2014), and have been used in a variety of visual dysfunctions (Regan et al., 1994, 1998; Silveira et al., 2003; Silva et al., 2005; Costa et al., 2007; Rodrigues et al., 2007; Moura et al., 2008; Lacerda et al., 2012). They can also be used to investigate the perceptual interaction of color and luminance in the identification of objects (Souza et al., 2014), to estimate certain effects in normal trichromats, e.g., age effect (Paramei, 2012; Paramei and Oakley, 2014), the effect of binocular summation (Costa et al., 2006).

Many scientific or commercial tools for color vision evaluation use pseudoisochromatic stimuli (Hardy et al., 1954; Regan et al., 1994; Silveira et al., 2003; Ventura et al., 2003; Bailey et al., 2004; Mancuso et al., 2006; Rodrigues et al., 2007; Goulart et al., 2008, 2013). However, few studies have investigated the influence of the different parameters that define pseudoisochromatic stimuli on subject's color vision performance (Mollon and Reffin, 1989; Regan et al., 1994; Souza et al., 2014).

Mollon and colleagues (Mollon and Reffin, 1989; Regan et al., 1994) combined the Stilling (1877) principles of breaking target and field into many small patches and varying the luminance of the individual patches, with those of Chibret (1887) of dynamically and adaptively varying the chromatic difference of target and field along different directions in color space to develop the CCT, a widely used assessment of color discrimination thresholds. They used the Cartesian distance between two chromaticities in the CIE 1976 color space to represent color contrast between target and field of pseudoisochromatic stimuli and observed that the correct target discrimination decreased as the distance between target chromaticity and field chromaticity decreased. After a threshold criterion was reached, the staircase procedure was terminated, the color discrimination threshold was estimated at different chromatic axes, and the MacAdam color discrimination ellipses were determined. Their results from congenital color blind subjects showed that color discrimination thresholds were increased along the specific confusion lines of their phenotypes.

Recently, Souza et al. (2014) investigated the influence of the number of luminance levels in the luminance noise of pseudoisochromatic stimuli on color discrimination thresholds. They observed that color discrimination thresholds exponentially decayed as a function of the number of luminance levels on the luminance noise. They observed that the greater the number of local areas with luminance contrast below 18.6% of Weber contrast, the higher were the color discrimination thresholds. Their results showed that the correct description of different features of pseudoisochromatic stimuli could be useful for comparisons between different experiments using this particular kind of stimulus.

Although luminance noise is a fundamental feature of pseudoisochromatic stimuli, its effect on chromatic discrimination is not clear. Therefore, the current study investigated the influence of the luminance contrast in the luminance noise as a function of mean luminance on chromatic discrimination, as measured by thresholds and reaction times (RTs) to suprathreshold stimuli.

Here, we investigated which feature of the luminance noise has more influence on the color vision perception, the mean luminance or how the noise luminance was modulated.

## Methods

This study comprised two different experimental paradigms to determine the influence of the luminance noise modulation on the color vision performance: color discrimination thresholds measurements and RT measurements. The luminance noise was modulated as a function of mean luminance in two ways: keeping constant the absolute luminance difference between the maximum and minimum luminance—constant delta protocol (CDP); or keeping constant the contrast between the maximum and minimum luminance—constant contrast protocol (CCP).

### Experiment 1: Color discrimination threshold measurements

#### Subjects and apparatus

Nine naïve subjects (25 ± 8 years old) participated in Experiment 1 measuring color discrimination thresholds. All subjects were personally invited and gave written consent to participate in the study. This study followed the tenets of the Declaration of Helsinki and it was also approved by the Ethical Committee for Research with Humans, Tropical Medicine Nucleus, Federal University of Pará, Brazil (Report #570.434) as well as the University of Tennessee Health Science Center IRB, Memphis, TN, USA. All subjects had no history of ophthalmological, neurological, or systemic diseases that would potentially impair their vision. The subjects had normal or corrected to 20/20 or better visual acuity, and normal color vision as evaluated by Ishihara's plate test and Farnsworth-Munsell 100 Hue test. For color discrimination threshold measurements, subjects were monocularly tested using the eye with the highest Snellen visual acuity.

The Cambridge Color Test (CCT, Cambridge Research System, CRS, Rochester, England, UK) was used for color discrimination threshold measurements. Pseudoisochromatic stimuli were generated in a ViSaGe platform (CRS) and displayed in a 21″ CRT monitor (Mitsubishi, Tokyo, Japan) at high spatial (1600 × 1200 pixels), temporal (125 Hz vertical frame rate), and chromatic resolution (14 bits per gun). Gamma-correction was used to calibrate the monitor using a ColorCal colorimeter (CRS). The CIE 1976 chromaticity coordinates of the stimulus field were *u'* = 0.1977, *v'* = 0.4689. In the stimuli, a “C” target, measuring 1° in the gap, 4.3° in the outer diameter, and 2.2° in the inner diameter, differed from the field by its chromaticity, which was changed along eight chromatic axes irradiating from the field chromaticity in the CIE 1976 color space. Subjects were placed in a dark room at 3.1 m away from the display. The stimulus presentation lasted 3 s.

#### Procedure

**Figure 1:**
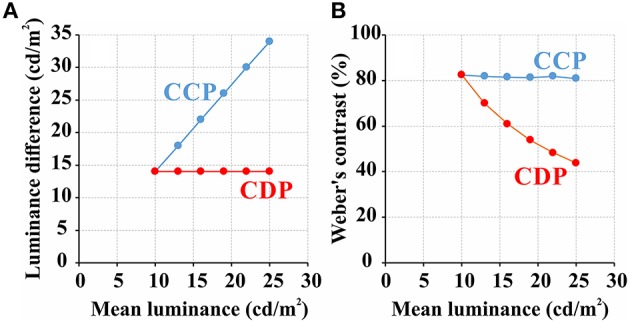
**Representation of the two forms of luminance contrast modulation used in this work**. There was a trade-off between absolute luminance difference and Weber's contrast between the maximum and minimum luminance of the luminance noise in the pseudoisochromatic stimuli used in this work. The absolute difference between maximum and minimum luminance of the luminance noise could be kept constant at different stimulus mean luminance (red line in **A**, CDP), but in this case the Weber's contrast decreased as a function of mean luminance (red line in **B**, CDP). On the other hand, when the luminance modulation was set to conserve the Weber's contrast constant (blue line in **B**, CCP), the absolute difference between the maximum and minimum luminance of the luminance noise increased as a function of mean luminance (blue line in **A**, CCP).

In the CDP the contrast between the maximum and minimum luminance of the luminance noise decreased as a function of the noise mean luminance, while in the CCP the absolute difference between the maximum and minimum luminance of the luminance noise increased as a function of the noise mean luminance. Figure 1 illustrates the differences between the two experimental protocols.

Color discrimination thresholds were estimated using the CDP and CCP modes of luminance contrast modulation of the luminance noise. For each protocol, six stimuli mean luminance were used: 10, 13, 16, 19, 22, and 25 cd/m^2^. In the CDP, the absolute difference between the maximum luminance and the minimum luminance of the luminance noise was 14 cd/m^2^ (7 cd/m^2^ above and below the mean luminance). In the CCP, the Weber's contrast of the luminance noise remained between 80.9 and 82.4%. Equations 1 and 2 were used to calculate the absolute difference and Weber's contrast for both protocols, CDP and CCP (see below). Table 1 shows the values of maximum luminance, minimum luminance, absolute difference, and Weber' contrast of the luminance noise at each stimulus mean luminance for each test protocol.

(1) $$\begin{array}{l} D=MaxLum-MinLum \end{array}$$

(2) $$\begin{array}{l} C=\frac{MaxLum-MinLum}{MaxLum} \end{array}$$

where *D* is the absolute difference, *C* is the Weber's contrast, *MaxLum* is the maximum luminance of the luminance noise, and *MinLum* is the minimum luminance of the luminance noise.

**Table 1:** **Contrast modulation of the luminance noise used in this study**.

**CONSTANT DELTA PROTOCOL (CDP)**				
**Mean luminance (cd/m^2^)**	**Maximum luminance (cd/m^2^)**	**Minimum luminance (cd/m^2^)**	**Absolute luminance difference (cd/m^2^)**	**Weber's contrast (%)**
10	17	3	14	82.4
13	20	6	14	70
16	23	9	14	60.9
19	26	12	14	53.8
22	29	15	14	48.3
25	32	18	14	43.8
**CONSTANT CONTRAST PROTOCOL (CCP)**				
10	17	3	14	82.4
13	22	4	18	81.8
16	27	5	22	81.5
19	32	6	26	81.3
22	37	7	30	81.8
25	42	8	34	80.9

Each subject previously adapted to the stimulus for 5 min. The subject was asked to identify the gap orientation selecting four possible alternatives: up, down, right, or left. A four-button box response (CB4, CRS) was used to record the subject's choice. A four-alternative forced-choice procedure was used, and a staircase method controlled stimulus presentation, modulating the vector distance between target and field chromaticities. Stimulus presentation ended after 12 reversals of the staircase and the color discrimination threshold was taken as the average of the last eight reversals.

In order to quantify the color discrimination thresholds, ellipse functions were fitted to the data points representing the eight-color discrimination thresholds measured. Ellipse fitting was performed using the Khachiyan ellipsoid method (Khachiyan, 1979) implemented with Matlab R2013a (Mathworks, Natick, Massachusetts, USA) routines. Ellipse areas were estimated for each stimulus condition and compared using one-way ANOVA and Tukey *post-hoc* test (*p* = 0.05).

In order to quantify the variation rate of ellipse area as a function of the stimulus mean luminance, the exponents of exponential functions fitted to the data points using the least square method were used (Equation 3). Student *t*-test (*p* = 0.05) was then used to compare exponent values obtained by using both protocols, CDP and CCP.

(3) $$\begin{array}{l} y=ae^{bx} \end{array}$$

Where *y* was the exponential model, *a* and *b* were free parameters and *e* was the number of Euler.

### Experiment 2: Reaction times measurements

#### Subjects and apparatus

Three subjects (37 ± 11 years old) participated in Experiment 2 measuring RTs to suprathreshold stimuli; two of these, #1 and #2, were experts in visual psychophysics, while #3 was naïve. For RT measurements, subjects were binocularly tested. During the experimental procedure, the pupils were in their natural state with no topical drugs applied.

All subjects were personally invited and gave written consent to participate in the study. This study followed the tenets of the Declaration of Helsinki. All subjects had no history of ophthalmological, neurological, or systemic diseases that would potentially impair their vision. The subjects had normal or corrected to 20/20 or better visual acuity, and normal color vision as evaluated by Ishihara's plate test and Farnsworth-Munsell 100 Hue test.

A CCT-adapted test was programmed in Matlab R2013a language using extensions provided by the Psychophysics Toolbox (Psychtoolbox 3; Kleiner, 2010). Pseudoisochromatic stimuli were shown in a high spatial, temporal, and color resolution LG ez T710SH 17″ CRT monitor (H-Frequency: 30~71 KHz; V-Frequency: 50–160 kHz; SXGA 60 Hz, 1280 × 1024 pixels; LG Electronics, Seoul, South Korea). Prior to the beginning of experiments, gamma correction was applied to the monitor using a CS-100A Chroma Meter (Konica Minolta, Tokyo, Japan).

As in the color discrimination threshold measurements (see previous section), a Landolt “C” was also employed as the stimulus target, differing from the field by its chromaticity, and having the same dimensions as in the previous experiment (4.3, 2.2, and 1° for outer diameter, inner diameter, and gap). The field chromaticity in the CIE1976 color space was *u'* = 0.143, *v'* = 0.465, and the target chromaticity varied along four chromatic axes (0, 90, 180, and 270°) from the field chromaticity.

#### Procedure

Each subjects were placed in a dark room at 1.3 m away from the display. The “C” gap was shown in two alternate orientations (right or left).

RTs were measured for the two cardinal chromatic axes of the MBDKL space and four directions (L−M, M−L, S+, and S−). Along each half-axes, 11 vector lengths were tested for each observer. Note that (i) each vector length represented a suprathreshold stimulus: it corresponded to the chromaticity of the target that has been responded to 100% correctly; (ii) the latter implied that vector lengths varied between the observers. The chromatic coordinates are chosen according to the lines that isolate opponent mechanisms called (L−M) and [S− (L + M)] in the DKL space. The chromatic coordinates of the stimulus are found on the line linking the white point with each of the points with (*u', v'*) coordinates. In this space: stimuli +L−M (0°) and -L+M (180°) (the angular azimuth in deg at MBDKL color space: (MacLeod and Boynton, 1979; Derrington et al., 1984)) are located on the axis that produces only excitation of the L- and M-cones -(L−M)-cone opponent mechanism. The axis corresponding to the [S− (L + M)]-cone opponent mechanism belongs to the conventional tritanopic line. The stimuli called +S (90°) and −S (270°) are located on this axis.

The locations of the maximum distances along each axis were: 0° axis, *u'* = 0.2125, *v'* = 0.4564; 90° axis, *u'* = 0.1604, *v'* = 0.3972; 180° axis, *u'* = 0.0735, *v'* = 0.4731; and for 270° axis, *u'* = 0.1250, *v'* = 0.5326.

All the procedures were performed using the two protocols described in the experiment 1, at each of three stimulus mean luminance: 13, 19, and 25 cd/m^2^. A two-alternative forced-choice task was used. To measure the RT, after stimulus presentation, the subject then pressed a two-button box (modified USB mouse) to indicate the “C” gap orientation (right or left) as soon as he/she had detected. Two-button box latency was measured resulting in a mean time latency of 40 ± 7 ms, for both buttons. This latency is the time from detection of gap, as a consequence of chromatic discrimination to the response, e.g., key press.

The stimulus conditions comprised mean luminance, chromatic axis, chromatic vector length, and luminance noise protocol. Within a block of trials, the mean luminance and then the chromatic axis were randomly selected. Once mean luminance and chromatic axes values were set, the observers were adapted to the mean luminance for 5 min prior to each block of trials. The other two stimulus parameters—luminance noise protocol and chromatic vector length—were randomly chosen from two different protocols of luminance noise and 11 chromatic vector lengths for each block of 5 trials. Each experimental session took one and a half hours. Each data point resulted from 50 trial measurements.

The stimulus presentation was preceded by a random time interval that varied between 1000 and 3000 ms, to prevent the subject from knowing when the stimulus would potentially appear. The time between the presentation of the stimulus and the observer's response was registered as the RT. The stimulus was turned off when the observer's response was recorded. Once the subject responded, the 1000–3000 ms random time interval was reintroduced and, during this period, the mean luminance was kept constant.

Only the correct positional responses were considered in the analysis of RT. The incorrect responses were also separately analyzed to determine which test protocol yielded more incorrect responses. To obtain results central tendency, cut-offs based on the interquartile range were used and values higher than the 75th percentile or lower than the 25th percentile of the distribution were discarded. Therefore, the mean RT was taken as a measure of the distribution central tendency. The mean RT and 95% confidence intervals were calculated for each experimental condition.

The fact that the experiment measuring thresholds was carried out monocularly and the experiment measuring RTs was carried out binocularly has no influence in our interpretation of the results. In the Brazilian laboratory where the color discrimination experiment was conducted, we used CCT for clinical evaluation, and all the experiments were carried out monocularly to be inserted in the database for healthy observers with normal color vision of the laboratory. In comparison, in the Argentinian laboratory where the RT experiment was conducted, these measurements were obtained binocularly, with no difference between the eyes mean (Jimenez et al., 2002). The RTs are longer monocular vision compared to binocular vision (Jimenez et al., 2002). Since in each experiment the focus was on the measurement change depending on variation of the luminance parameters, the stimulus presentation mode, monocular vs. binocular, is unlikely to affect the comparison of results between the two experiments.

Pieron (1952) showed that for a given perceptual modality, reaction time decreases as the stimulus intensity increases, following a power function. Reaction time is limited by a minimum value or asymptotic level (RT_0_). Following Piéron's model, Equation 4 shows color detection RT as a function of the reciprocal of chromatic vector length:

(4) $$\begin{array}{l} RT={RT}_{0}+\frac{k}{x^{n}} \end{array}$$

*RT* was the reaction time, *RT*_0_ was the asymptotic value of RT, *k* was the curve steepness, *x* represented the chromatic vector length expressed in the *u'v'* dimensions of the CIE1976 color space or the distance between the coordinates of the stimulus and field chromaticities, and *n* was the exponent.

According to Plainis and Murray (2000) and McKeefry et al. (2003), the Equation 4 exponent is equal to 1. If we assume that *n* = 1 then the relation between *RT* and *x* is linear. In this case, Equation 4 becomes:

(5) $$\begin{array}{l} RT={RT}_{0}+k.\;y \end{array}$$

Using *y* as the reciprocal of *x*.

## Results

### Experiment 1: Color discrimination thresholds

**Figure 2:**
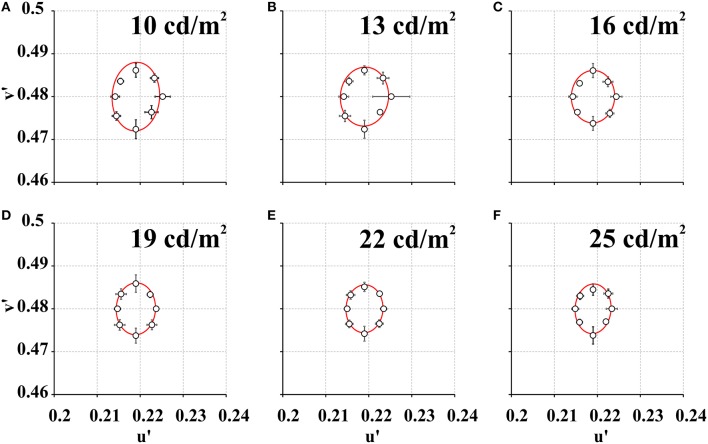
**Mean color discrimination ellipses estimated at a series of stimulus mean luminance using the CDP**. **(A)** 10 cd/m^2^, **(B)** 13 cd/m^2^, **(C)** 16 cd/m^2^, **(D)** 19 cd/m^2^, **(E)** 22 cd/m^2^, and **(F)** 25 cd/m^2^. For all subjects, ellipse area decreased as a function of the stimulus mean luminance. Ellipse area at 10 cd/m^2^ and 13 cd/m^2^ was statistically significantly larger than at 25 cd/m^2^. Empty circles represent mean color discrimination thresholds (*n* = 9) for eight different chromatic axes. Red curves represent the best ellipse fit to the color discrimination thresholds. Vertical and horizontal bars represent standard deviations in the *u'v'* coordinates of the CIE1976 color space.

Figure 2 shows mean color discrimination ellipses estimated using the CDP at six values of stimulus mean luminance. Table 2 shows the range and average for ellipse areas obtained with the CDP. Ellipse areas measured at 10 and 13 cd/m^2^ were statistically larger than ellipse area measured at 25 cd/m^2^ (*p* < 0.05). Ellipse areas of all subjects had an exponential decay as a function of the stimulus mean luminance (**Figure 4A**). The mean exponent of the exponential fit was −0.042 ± 0.023.

**Table 2:** **Ellipse area measured using either the CDP or CCP**.

**CONSTANT DELTA PROTOCOL (CDP)**			
**Mean luminance (cd / m^2^)**	**Largest ellipse area**	**Smallest ellipse area**	**Ellipse area mean ± standard deviation**
10	0.267	0.116	0.160 ± 0.04
13	0.347	0.057	0.149 ± 0.08
16	0.159	0.064	0.109 ± 0.035
19	0.181	0.068	0.105 ± 0.043
22	0.127	0.067	0.093 ± 0.02
25	0.132	0.048	0.088 ± 0.033
**CONSTANT CONTRAST PROTOCOL (CCP)**			
10	0.270	0.116	0.171 ± 0.061
13	0.221	0.102	0.142 ± 0.046
16	0.231	0.074	0.151 ± 0.057
19	0.207	0.068	0.125 ± 0.041
22	0.303	0.067	0.135 ± 0.072
25	0.164	0.032	0.108 ± 0.032

**Figure 3:**
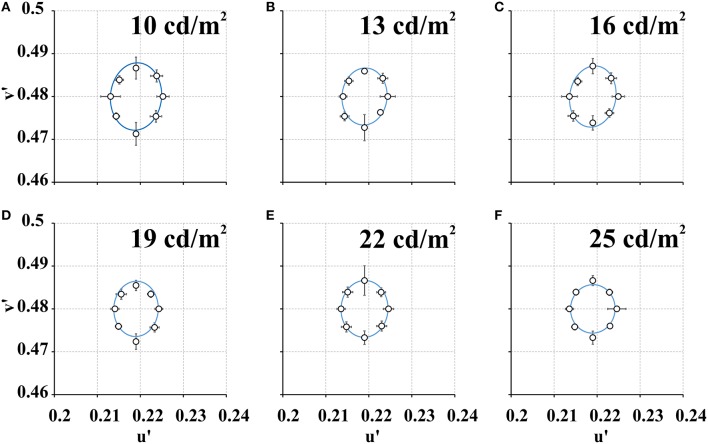
**Mean color discrimination ellipses estimated at a series of stimulus mean luminance using the CCP**. **(A)** 10 cd/m^2^, **(B)** 13 cd/m^2^, **(C)** 16 cd/m^2^, **(D)** 19 cd/m^2^, **(E)** 22 cd/m^2^, and **(F)** 25 cd/m^2^. There was no significant difference between ellipse areas measured at different stimulus mean luminance. Empty circles represent mean color discrimination thresholds (*n* = 9) for eight different chromatic axes. Blue curves represent the best fit to the color discrimination thresholds. Vertical and horizontal bars represent standard deviations in the *u'v'* coordinates of the CIE1976 color space.

Figure 3 shows the mean color discrimination ellipses obtained with the CCP at the six values of stimulus mean luminance. Table 2 shows the range and average for ellipse areas obtained with the CCP. There were no significant between ellipse areas measured at the six stimuli mean luminance (*p* > 0.05). Most of subjects had an exponential decay of their ellipse areas as a function of the stimulus mean luminance but two of them had positive exponents instead in the exponential fitting. The mean exponent of the exponential fit was −0.025 ± 0.023. When the exponents of the exponential functions of individual fittings were compared, the decay was shown to be steeper in the CDP than CCP (*p* < 0.05).

**Figure 4:**
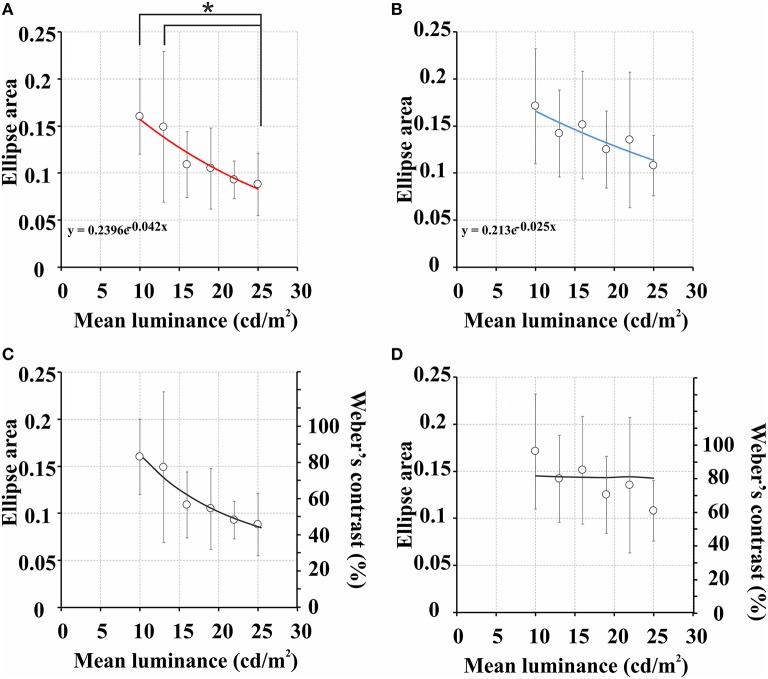
**Areas of color discrimination ellipses as a function of stimulus mean luminance. (A)** Data obtained with the CDP. **(B)** Data obtained with the CCP. The functions were fitted with exponential functions (red and blue lines in **A** and **B**, respectively) and the exponent was use as the parameter to express ellipse area decay with stimulus mean luminance. Functions estimated with CDP had exponent significantly higher than functions estimated with CCP (−0.044 ± 0.023 vs. −0.025 ± 0.023, respectively). In **(C)** and **(D)**, data points from **(A)** and **(B)** were fitted with functions representing the differential change in the Weber contrast with the stimulus mean luminance that occur when using either the CDP or CCP, respectively (black lines); see Figure 1 for details. Empty circles and vertical bars represent mean ellipse areas and standard deviations. ^*^Statistical significant differences using one-way ANOVA test, Tukey *post-hoc* test, *p* < 0.05.

Figures 4A,B shows mean ellipses areas as a function of stimulus mean luminance for each test protocol. The plots showed that color discrimination thresholds decreased with the mean luminance of the stimulus. Figures 4C,D shows both series of data points fitted to the functions describing the change of Weber's contrast with stimulus mean luminance for each test protocol (Figure 1). In the CDP (Figure 4C), increasing stimulus mean luminance led to an exponential decay of the Weber's contrast of the luminance noise with an exponent of 0.042, which was very similar to the exponent of the mean function (−0.044 ± 0.023). In the CCP (Figure 4D), although the ellipse area showed a smaller exponential decay as a function of the stimulus mean luminance (mean exponent = −0.025 ± 0.023), in comparison with the CDP, data points were still well-described by flat Weber's contrast for the stimulus condition used.

### Experiment 2: Reaction time measurements

**Figure 5:**
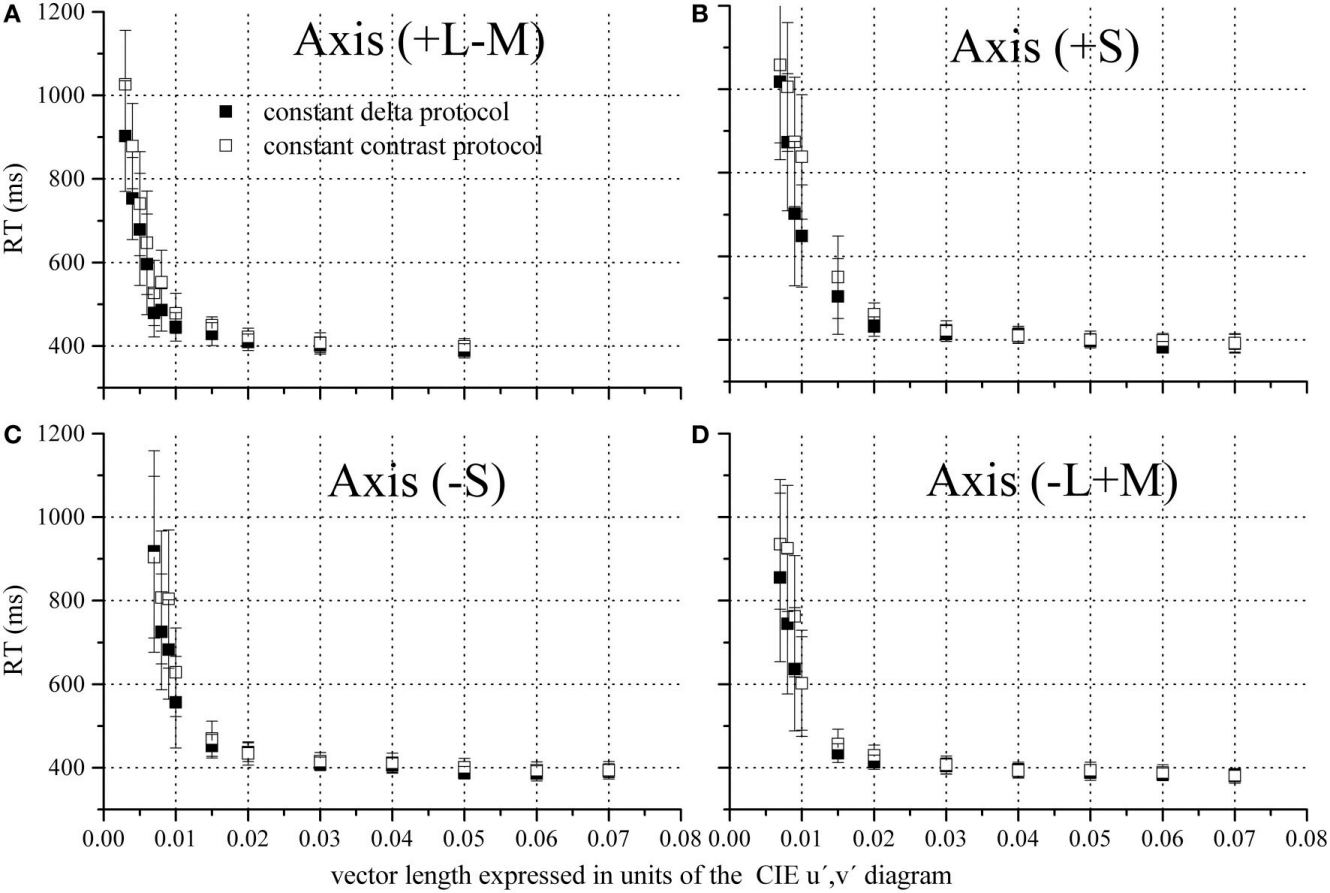
**Reaction time as a function of the chromatic vector length**. Four chromatic axes were studied, irradiating from the field chromaticity in the CIE 1976 color space: **(A)** +L−M (0°), **(B)** +S (90°), **(C)** −L+M (180°), and **(D)** −S (270°). Only results corresponding to the mean luminance of 13 cd/m^2^. Reaction time became faster when vector length was increased for both test protocols, either CDP (filled squares) or CCP (empty squares), and its value decreased toward an asymptotic value. Squares and vertical bars represent means and standard deviations.

Reaction time as a function of the chromatic vector length for four chromatic axes and mean luminance 13 cd/m^2^ were shown in Figure 5. The RTs data corresponding to 19 and 25 cd/m^2^ show similar pattern as seen in Figure 5. Reaction time rapidly decreased with increasing chromatic vector length toward an asymptotic value for the four chromatic half-axes that were studied.

**Figure 6:**
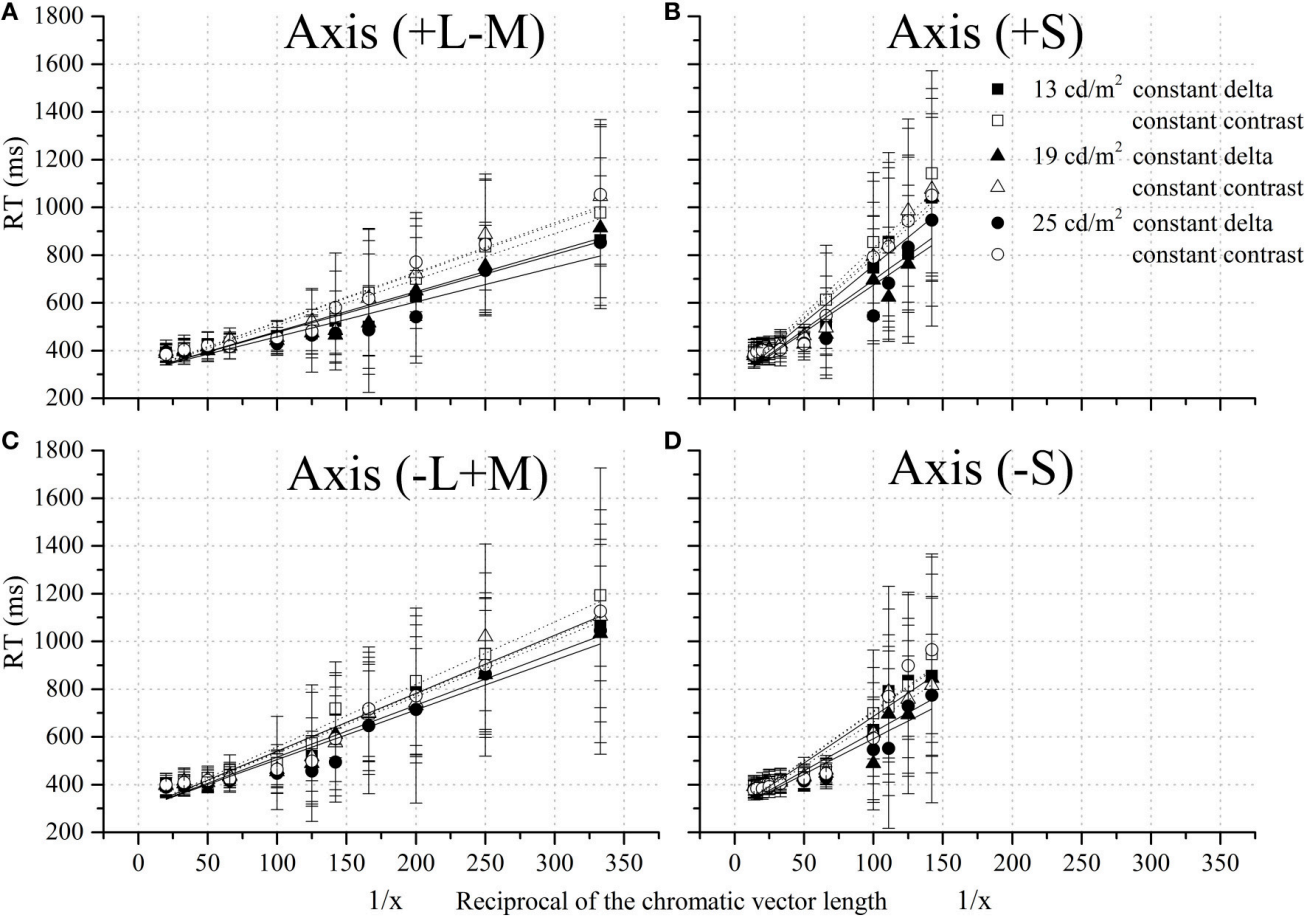
**Reaction time as a function of the reciprocal of the chromatic vector length at four chromatic axes**. Four chromatic axes were studied: **(A)** +L–M (0°), **(B)** +S (90°), **(C)** −L+M (180°), and **(D)** –S (270°). There was significant effect on chromatic axes (*p* < 0.05), test protocol (*p* < 0.05), and stimulus mean luminance (*p* < 0.05). At all chromatic axes, the reaction time was longer using the CCP than the CDP at the highest values of reciprocal of the chromatic vector length. Squares, triangles, and circles represent data points obtained at 13, 19, and 25 cd/m^2^. Filled symbols represent data points obtained with the CDP, while empty symbols represent data points obtained with the CCP.

The linear relationship between RT and the reciprocal of chromatic vector length were shown in Figure 6 for the four chromatic half-axes studied. All data fit a straight line, according to Equation 5, whose slope depended on the stimulus parameters.

In order to know whether the RT has a linear relationship with the reciprocal of vector length according to Equation 5, RTs data for each observer were analyzed using analysis of covariance (ANCOVA) in a general linear model, with chromatic axis, noise test protocol, and mean luminance as categorical independent variables (factors); the reciprocal of chromatic vector length as a continuous predictor variable (covariate) and, RT as a dependent variable. The results of this analysis showed a significant effect of the reciprocal of chromatic vector length on the RTs [*F*_(1, 13073)_ = 13844, *p* < 0.05 for Subject #1; *F*_(1, 12972)_ = 121.6, *p* < 0.05 for Subject #2; *F*_(1, 11388)_ = 2904, *p* < 0.05 for Subject #3], which demonstrates that the chromatic vector length has a linear relation with the RT. In addition, the test of SS whole model as a function of SS residual provided a value of multiple R equal to 0.72 for Subject #1, 0.70 for Subject #2, and 0.48 for Subject #3. Hence, performance measured by RTs obeyed the Pieron's law with an exponent equal to 1.

To look at the dependence of RTs with the other factors considered in this study, the data were also analyzed as a function of the reciprocal of the chromatic vector length. The statistical analysis (multivariate ANOVA with four factors) showed a significant effect of the factors: subjects [*F*_(2, 37435)_ = 6200.86, *p* < 0.05], chromatic axes [*F*_(3, 37435)_ = 2019.80, *p* < 0.05], test protocols [*F*_(1, 37435)_ = 464.74, *p* < 0.05], and stimulus mean luminance [*F*_(2, 37435)_ = 93.05, *p* < 0.05]. Variation of RTs among the subjects could be explained by the difference between expert Subjects #1 and #2 compared to naive Subject #3. Nevertheless, the results consistently showed similar patterns in the three cases. Reaction times were higher for the CCP than CDP, but this difference depended on the chromatic axes and stimulus mean luminance.

Following the Equation 5, RT data were fitted assuming a value of *n* = 1 and the asymptotic value *RT*_0_ for each experimental condition for each subject was estimated. The values of *RT*_0_ were submitted to ANOVA, which showed that *RT*_0_ depended on the subject [*F*_(2, 63)_ = 166.067, *p* < 0.05] and chromatic axis [*F*_(3, 63)_ = 18.877, *p* < 0.05], but did not depend on the noise test protocol or the stimulus mean luminance. A *post-hoc* Tukey analysis showed no statistical significant difference between +L−M (0°) and −L+M (180°) axes (Approximate probabilities for *post-hoc* tests *p* > 0.05) or between +S (90°) and −S (270°) axes (Approximate probabilities for *post-hoc* tests *p* > 0.05).

**Figure 7:**
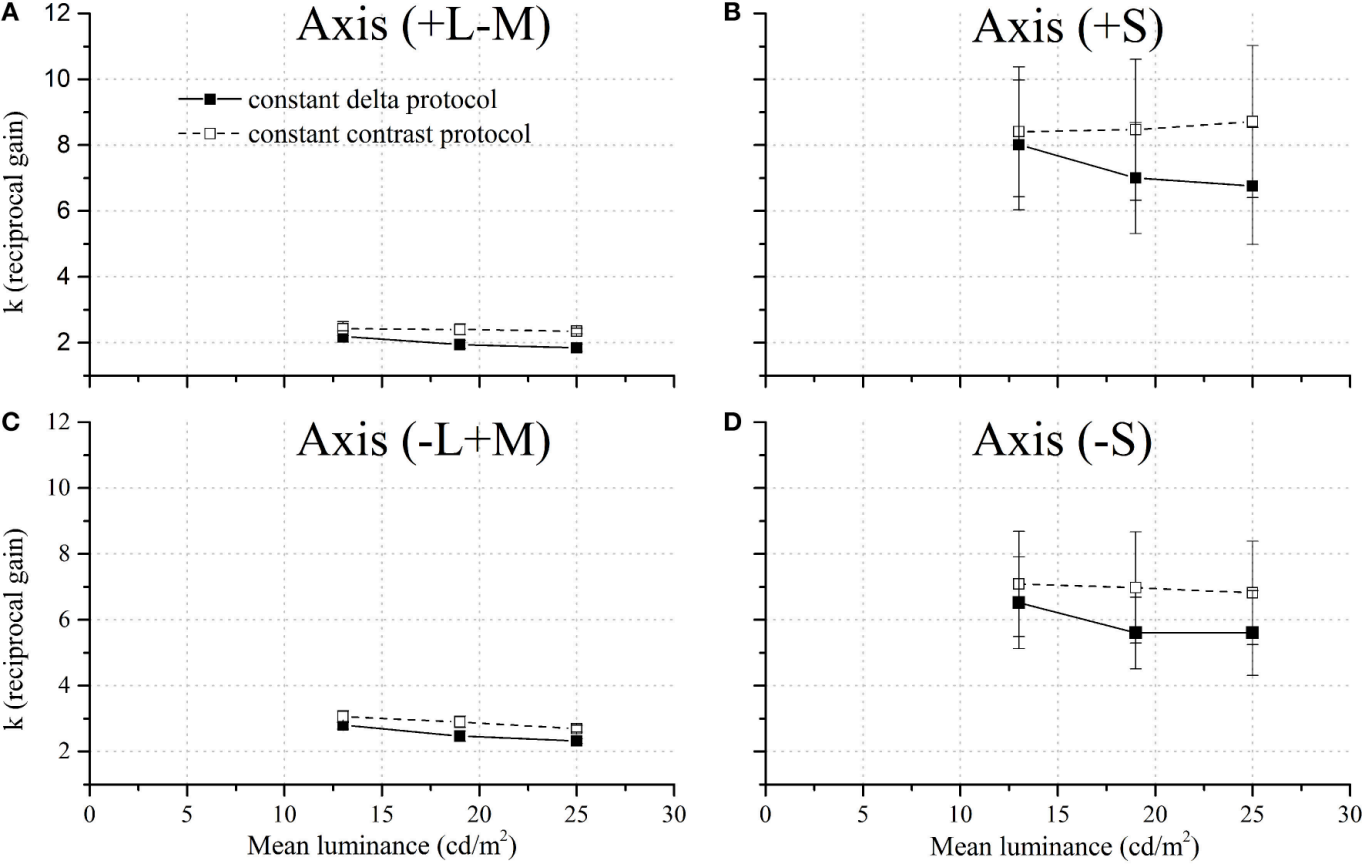
**k- reciprocal gain—as a function of stimulus mean luminance at four chromatic axes**. **(A)** +L−M (0°), **(B)** +S (90°), **(C)** −L+M (180°), and **(D)** −S (270°). In general, the reciprocal gain showed a steeper decay with the stimulus mean luminance for the CDP (filled squares) than CCP (empty squares). Axes located in the color space along the two half-axes: +S (90°) and −S (270°) showed more clearly this effect than axes located in the two half-axes: +L−M (0°) and −L+M (180°).

The values of *k*—the reciprocal of the gain—were obtained using fixed *n* and *RT*_0_–calculated before-and they are shown in Figure 7. Data showed that *k* was lower (faster RTs) for CCP than for CDP (slower RTs) for stimuli modulated along −S (270°) and +S (90°) chromatic half-axis. These differences were more marked for the highest mean luminance. Nevertheless, the gain for stimuli modulated along +L−M (0°) and −L+M (180°) remains constant.

The values of *k* were subsequently subjected to ANOVA with four factors. This analysis confirms what is previously stated: the values of *k* varied significantly with chromatic half-axis [*F*_(3, 63)_ = 79.4580, *p* < 0.05], subject [*F*_(2, 63)_ = 32.2156, *p* < 0.05], and noise test protocol [*F*_(1, 63)_ = 6.0432, *p* < 0.05], but no statistical significance was observed for the mean luminance of the noise (*p* > 0.05). A Tukey post ANOVA test showed no significant differences between CDP and CCP for stimuli along +L−M (0°) and −L+M (180°) chromatic axes. Significant differences between CDP and CCP were observed for stimuli along +S (90°) and −S (270°) chromatic axes.

**Figure 8:**
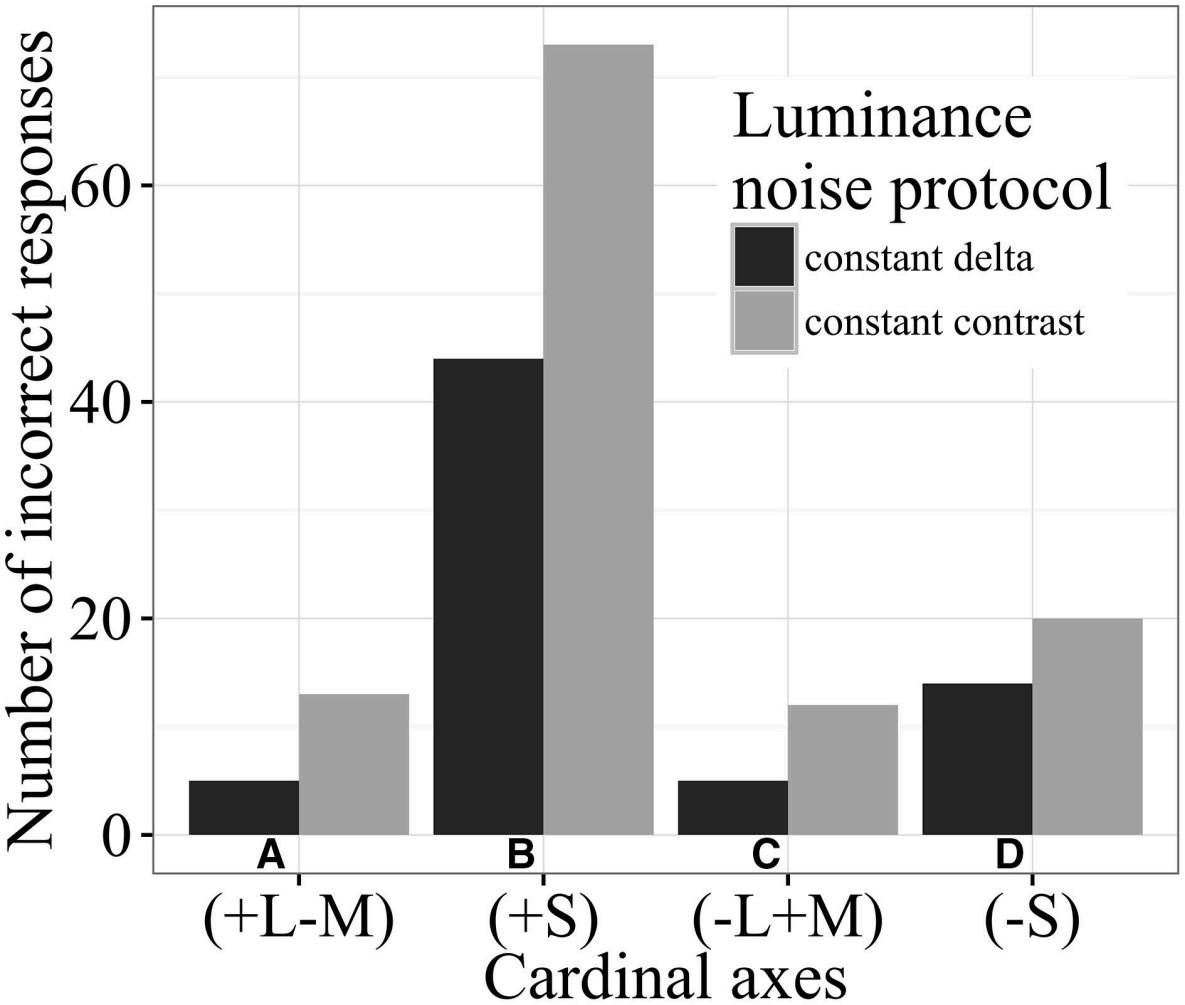
**Number of incorrect responses in the reaction time experiments using different luminance noise protocols**. Data from Subject #2 for CDP and CCP for stimuli located along the four chromatic axes: **(A)** +L−M (0°) **(B)** +S (90°), **(C)** −L+M (180°), and **(D)** −S (270°). Field luminance was 13 cd/m^2^. The results depended on the test protocol, with a larger number of incorrect for CCP than CDP. The number of incorrect responses also depended on the chromatic axes, being larger for +S (90°) and −S (270°) than for +L−M (0°) and −L+M (180°). The results for the other two subjects tested and field luminance values were similar.

Figure 8 illustrates the number of incorrect responses made by subjects using CDP or CCP. Only data obtained from Subject #2 are shown, but the results from the other two subjects were similar. The number of incorrect responses was larger for CCP when compared with CDP experiments. The number of incorrect responses also depended from the chromatic axis used: number of incorrect responses were larger along +S (90°) and −S (270°) than +L−M (0°) and −L+M (180°). Possibly the greater number of incorrect responses for the (+S) half-axis was caused by variation of the chromaticity within the metric framework of the CIE 1976 diagram: the latter is not fully uniform, with the difference in the length of MacAdam ellipses varying 1:4 between the upper part [~ (−S)] and the lower part [~(+S)] of this chromaticity diagram. So, it is possible that when variation in stimulus chromaticity along the (+S) half-axis in *u'v'* coordinates was comparable to that of (−S) half-axis, in subjective terms the degrees of excursion were far from being comparable.

## Discussion

Pseudoisochromatic stimuli are simple, interesting patterns created in laboratory and used to simulate objects observed in a natural setting. They are composed by patches with variable color and luminance forming a target embedded in a field that differ from each other only by their color—the spatial and luminance noise assure that color discrimination is essential for detection and identification of the target.

Few investigations have focused on the influence of stimulus parameters of pseudoisochromatic patterns on the color visual performance, such as the range of luminance noise, spatial noise and number of spatial patches (Regan et al., 1994; Souza et al., 2014). Luminance noise has an important influence on subjects' performance to discriminate the target of pseudoisochromatic patterns and some studies have reported with details how the luminance noise of pseudoisochromatic stimuli was modulated in their experimental paradigms (Regan et al., 1994; Goulart et al., 2013; Souza et al., 2014). In the work of Regan et al. (1994), the luminance of any given patch varied from trial to trial and was randomly assigned to one of six equally spaced and equally probable levels in the range 7.6−17.0 cd/m^2^. In the case of Goulart et al. (2013), the stimulus arrangement, occupying the entire screen, was composed of circles of different sizes at six levels of luminance that randomly varied between 7 and 15 cd/m^2^ and, finally for Souza et al. (2014), the minimum and maximum luminance values of the luminance noise were 8 and 18 cd/m^2^, respectively, with Weber's contrast of the noise about 53−55%. In all cases, they use a single value of mean luminance. The thresholds at 22 and 25 cd/m^2^ (or at least 25 cd/m^2^) obtained in this study are lower than thresholds in the Regan et al. (1994), Goulart et al. (2013), and Souza et al. (2014) studies whose highest mean luminance was at 17, 15 and 18 cd/m^2^, respectively. Since the Weber's contrast in the three named studies was 0.53–0.55, i.e., lower than 80% in the CCP here, in line with the present main finding that the lower the contrast, the lower the thresholds. In these former studies the reported thresholds are expected to be lower than in the CCP, but comparable to the CDP at the mean luminance 16 cd/m^2^ (contrast of 0.61) or 19 cd/m^2^ (contrast of 0.54).

This was the first study to focus on the influence of the way how luminance modulation was applied to the luminance noise on the discrimination of pseudoisochromatic stimuli. Two different forms of luminance noise modulations were studied, either keeping constant the absolute difference between the maximum and minimum luminance and allowing the Weber's contrast to change (CDP) or keeping the Weber's contrast constant and allowing the absolute difference between the maximum and minimum luminance of the noise to change (CCP).

The results indicated that subjects' response to pseudoisochromatic stimuli depended not only of the chromatic information present in the pattern but also of the protocol that was used to create the luminance noise. Switkes et al. (1988) investigated the effects of luminance masking on color test reduced the color contrast threshold detection if the luminance contrast of the masking is lower, but the masking at high luminance contrast increased the threshold of color contrast detection. They indicated that the reduction of the color contrast detection only happen when the luminance masking had been about 32 times its threshold. They suggested that a mechanism that would produce these effects, would be a direct and attenuated input from luminance system on the chromatic system, and that the attenuation would reflect the lower contrast sensitivity of the color detection mechanism (P pathway) compared to the mechanism that process the luminance contrast threshold.

It was previously observed that at low mean luminance levels, color discrimination became poorer than at high levels (MacAdam, 1942; Brown, 1951; Wyszecki and Fielder, 1971). These previous studies used a larger range of mean luminance values than was the case of the current work. So, in the present study, we found for the CDP a decrease of the color discrimination as a function of the mean luminance in a very small range of luminance, significantly larger at the two lowest mean luminances of the noise, 10 cd/m^2^ and 13 cd/m^2^, compared to the highest one, 25 cd/m^2^. Nevertheless, for CCP noise protocol we found no significant difference between the values obtained within the mean luminance range that we studied.

We used a range of mean luminance inside the photopic range and the maximum and minimum values differ from each other by slightly more than one log unit. Although in the literature, there is no definite indication of the luminance boundary between the mesopic and the photopic range, probably only the stimulus with mean luminance of 10 cd/m^2^ is on the upper border of the mesopic range. As we found difference between conditions of a mixture of mesopic and photopic luminances (lowest mean luminance conditions) and photopic conditions (highest mean luminance condition), we could hypothesize that the results could have influence of the contribution of rods to the color perception (Zele and Cao, 2015). Rods influence on the color perception decreasing saturation of spectral lights and improves discrimination at long wavelengths or impair the tritan axis ordering hues (Stabell and Stabell, 1977; Buck et al., 1998; Knight et al., 1998). We thought that probably our results cannot be explained by rods influence on the color perception, because it occurred only for one of the tested protocols (CDP), we have measured the color discrimination on the threshold level, we found no specific improvements or impairments of the color discrimination, and we used only the foveal vision.

When the difference between the maximum luminance and minimum luminance present in the luminance noise was kept constant (CDP) across the range of mean luminance used, subject's performance—chromatic discrimination and RTs—improved with the mean luminance, following the Weber's contrast. In this case as the Weber's contrast of the noise decreases, subject's visual performance increases, reflecting the fact the Weber's contrast of the noise is a relevant parameter. In CDP protocol case, the visual response also depends on the chromaticity of the stimulus: when the stimulus was modulated on the (L−M) chromatic axis the response was independent of the mean luminance, not so in the case of stimuli modulated on the [S− (L + M)] chromatic axis. Meanwhile, when the Weber's contrast of the luminance noise was kept constant (CCP test protocol), subject's visual performance remained constant throughout the range of mean luminance tested with stimuli modulated either on (L−M) or [S− (L + M)] chromatic axes. The differences between RTs data for the two luminance noise protocol (CDP and CCP) were significantly lower at the lowest mean luminances of the noise, 13 cd/m^2^, compared to the highest one, 25 cd/m^2^. It seems that as the mean luminance of the noise decreases the dependence on the luminance noise protocol is not significant.

The range of Weber's luminance contrast of the noise used in this work was relatively high, between about 40 and 85%, well inside the dynamic range of visual pathways that combine low luminance contrast sensitivity with high color contrast sensitivity, such as the P or K pathways. As suggested by Souza et al. (2014), P pathway could be an adequate candidate to integrate luminance contrast information and color contrast information in the perception of pseudoisochromatic stimuli, such as those used in the current study, since P cells are very sensitive to red-green contrast and few sensitive to luminance contrast (Kaplan and Shapley, 1986; Lee et al., 1989a,b; Lee et al., 1990, 2011), as well as K cells that decode blue-yellow information and can also contribute to the luminance perception (Ripamonti et al., 2009).

Independently of the mean luminance of the noise and noise protocol used there were differences in gain, between the (L−M)- and S-opponent mechanisms: greater for stimulus modulated along +L−M (0°) and −L+M (180°) chromatic axes than for stimuli modulated along [S− (L + M)] chromatic mechanism. At the same time the gain is greater for −S (270°) stimuli than for +S (90°) stimuli. This result is not new and are in agreement of previous reports by Parry et al. (2004) and O'Donell et al. (2010). Sankeralli and Mullen (2001) found that the two opposing submechanisms +L−M (0°) and −L+M (180°) possess a close degree of symmetry in the weighting of their cone inputs. In comparison, RTs generated in response to −S stimuli also tended to be shorter than for +S stimuli at equal multiples above detection threshold. Moreover, they confirm other previous results that tritan system was more sluggish than the L/(L+M) system (e.g., Smithson and Mollon, 2004; Bompas and Sumner, 2008).

On the other hand, the chromatic discrimination threshold and RTs times represent different behavioral measures: both can be obtained in the same (detection to discrimination) task but the former refers to discriminability of the stimuli, whereas the latter to the chronology of discrimination. The two functions reflect change in luminance (achromatic) contrast in a similar way but are not identical (see e.g., Tiippana et al., 2001). Although, the discrimination threshold and RT are different performance measures, the results presented here show that the spatial luminance noise affects both in similar ways: RTs and color discrimination thresholds were dependent of the Weber's luminance contrast between maximum and minimum luminance of the luminance noise.

It is clear from the above stated that in order to compare results of different studies is important to specify how the luminance noise was generated and, if it has been created according a protocol like CDP we should indicate the condition of the stimulus presented

Finally, we think that other aspects of the luminance noise on the color vision perception can be questioned in future investigations, such as how the existence of the luminance noise on pseudoisochromatic stimulus can influence on the color discrimination or in the target detection. Other aspects of the luminance noise in pseudoisochromatic stimuli that potentially influence chromatic discrimination and, hence, segregation of the target from the field, can be investigated in the future, such as greater range of mean luminances and/or of luminance contrast.

## Author contributions

GS, BO, IC, AM, LS, BG: Conception and design of the main idea. BO, IC, AM: Conception and design of the reaction time experiments. GS, LS, BG: Conception and design of the color discrimination threshold experiment. BO, IC, AM: Acquisition, analysis and interpretation of data from reaction time measurements. GS, TC, EL, MJ: Acquisition, analysis and interpretation of data from color discrimination threshold measurements. GS, BO, IC, AM, TC, EL, MJ, DV, MF, LS: Drafting and revising the work for important intellectual content. GS, BO, IC, AM, TC, EL, MJ, DV, MF, LS, BG: Final approval of the version to be published. GS, BO, IC, AM, TC, EL, MJ, DV, MF, LS, BG: Agreement to be accountable for all aspects of the work in ensuring that questions related to the accuracy or integrity of any part of the work are appropriately investigated and resolved.

### Conflict of interest statement

The authors declare that the research was conducted in the absence of any commercial or financial relationships that could be construed as a potential conflict of interest.
